# Long noncoding RNA lncARSR promotes nonalcoholic fatty liver disease and hepatocellular carcinoma by promoting YAP1 and activating the IRS2/AKT pathway

**DOI:** 10.1186/s12967-020-02225-y

**Published:** 2020-03-13

**Authors:** Yuan Chi, Zheng Gong, He Xin, Ziwen Wang, Zhaoyu Liu

**Affiliations:** Department of Radiology, Shengjing Hospital of China Medical University, No. 36, Sanhao Street, Heping District, Shenyang, 110004 Liaoning People’s Republic of China

**Keywords:** Nonalcoholic fatty liver disease, Hepatocellular carcinoma, lncARSR, YAP1, IRS2, AKT

## Abstract

**Background:**

Nonalcoholic fatty liver disease (NAFLD) is the main cause for hepatocellular carcinoma (HCC). This study was intended to identify the function of long non-coding RNA (lncRNA) lncARSR in NAFLD and its role in human HCC cells (HepG2) proliferation and invasion.

**Methods:**

LncARSR expression was detected both in high fatty acid-treated HepG2 cells and NAFLD mouse model. After gain- and loss-of-function approaches in high fatty acid-treated HepG2 cells and NAFLD mice, lipid accumulation in livers from NAFLD mice and high fatty acid-treated cells was determined by H&E staining, Oil Red-O staining or Nile Red staining respectively. Expression of YAP1, adipogenesis- (Fasn, Scd1 and GPA) and IRS2/AKT pathway-related genes was measured. Cell proliferation was monitored by MTT and soft-agar colony formation assays, cell cycle was analyzed by flow cytometry, and cell invasion was examined by transwell assay. The tumor weight and volume were then measured through in vivo xenograft tumor model after silencing lncARSR.

**Results:**

LncARSR was highly expressed in high fatty diet (HFD)-fed mice and high fatty acid-treated HepG2 cells. LncARSR was observed to bind to YAP1, which inhibited phosphorylation nuclear translocation. LncARSR activated the IRS2/AKT pathway by reducing YAP1 phosphorylation, and further increased lipid accumulation, cell proliferation, invasion and cell cycle. Silencing lncARSR in HFD-fed mice alleviated NAFLD by regulating YAP1/IRS2/AKT axis.

**Conclusion:**

Silencing lncARSR suppressed the IRS2/AKT pathway, consequently reducing HCC cell proliferation and invasion and inhibiting lipid accumulation in NAFLD mice by downregulating YAP1, which suggests a clinical application in treating NAFLD.

## Background

Nonalcoholic fatty liver disease (NAFLD) is a chronic liver disease commonly seen in over-weight or obese people. Almost 90% of obese people have been diagnosed with diseases associated with fatty liver [1]. As the obesity epidemic grows, the incidence of NAFLD is accordingly increasing [2]. Besides, NAFLD has become the leading cause for liver cancer [3]. Therefore, efficiently treating NAFLD will be beneficial to prevent hepatocellular carcinoma (HCC). However, limited investigations on the mechanisms underlying the development of NAFLD have been reported [4].

Long non-coding RNAs (lncRNAs) may act as potential markers for prognosis and progression of liver diseases and furthermore as direct targets for therapeutic purposes. Several lncRNAs have been proved to be associated with liver diseases [5]. LncRNA activated in renal cell cancer (RCC) with Sunitinib Resistance (lncARSR), which is located on chromosome 9q82, was found to be up-regulated in sunitinib resistance of RCC [6]. According to a prior study [7], lncARSR is also potentially involved with hepatic steatosis. However, the role of lncARSR in NAFLD is less clear.

Yes-associated protein (YAP) is an effective transcriptional co-activator in the Hippo pathway, which is associated with the regulation of organ size through mediating cell proliferation, cell cycle and apoptosis. A higher level of YAP has frequently been detected in various types of human cancers [8, 9]. Moreover, the AKT pathway was demonstrated to be activated by YAP [10]. Further investigation highlighted that the Hippo pathway interacts with the AKT pathway by influencing insulin receptor substrate 2 (IRS2) expression in NAFLD, which affects the development of NAFLD and even HCC [11].

In this study, we investigated the effects of lncARSR/YAP1 on NAFLD, and finally identified that lncARSR promoted NAFLD through YAP1 and the IRS2/AKT pathway, thus providing a novel insight into NAFLD treatment.

## Materials and methods

### Ethical statement

All the animal experiments complied with the standard ethical guidelines prescribed in Guide for the Care and Use of Laboratory Animals by National Institutes of Health. All efforts were made to avoid unnecessary distress to the animals.

### Mouse model

A total of 60 C57BL/6 male mice (aged 6 weeks) were purchased from Beijing Huafukang Biotechnology Co., Ltd. (Beijing, China). The mice were fed with normal diet (carbohydrate accounted for 62.3% of total calories; fat 12.5%; protein 24.3%) or high fat diet (HFD) (carbohydrate accounted for 32.6% of total calories; fat 51.0%; protein 16.4%) (NAFLD mice). After 4 weeks, mice were euthanized by intraperitoneal injection of tripled 3% pentobarbital sodium (P3761, Sigma-Aldrich, St. Louis, MO, USA). The livers were separated for further analyses.

### Cell culture

The human HCC cells (HepG2) (http://www.cellbank.org.cn/) were cultured overnight in four-well chamber slides with Dulbecco’s modified eagle medium (DMEM) containing 10% fetal bovine serum (FBS). Then, the cells were cultured with DMEM supplemented with 1% w/v fatty acid-free bovine serum albumin (BSA) and 0.5 mM oleate. Meanwhile, HepG2 cells were infected with lentivirus of short hairpin (sh)-lncARSR, over-expression (oe)-lncARSR, sh-YAP1, oe-YAP1 or YAP1S127D (a phosphorylated mimic form of YAP1) singly or in combination. After 24 h of infection, lipid accumulation in HepG2 cells was determined by Nile Red Staining. The level of intracellular triglyceride (TG) was measured following the protocols of detection kits (Applygen, Beijing, China).

### RNA isolation and quantitation

Total RNA was extracted from cells and tissues with the Trizol kit (Thermo Fisher Scientific, Waltham, MA, USA) and then reversely transcribed into cDNA using the PrimeScript RT Reagent Kit (TaKaRa, Tokyo, Japan). Fluorescence quantitative polymerase chain reaction (qPCR) was subsequently carried out referring to the operation instruction provided by SYBR^®^ Premix Ex Taq™ II kit (Tli RNaseH Plus, TaKaRa) on Thermal Cycler Dice Real Time System (TP800, TaKaRa). The primers were synthesized by Guangzhou Ribobio Science & Technology Co., Ltd. (Guangzhou, China) (Table 1). Gene expression was measured by the 2^−ΔΔCt^ method with glyceraldehyde-3-phosphate dehydrogenase (GAPDH) as internal reference.

**Table 1:** Primer sequences for RT-qPCR

Genes	Primer sequences
lncARSR (Humo)	F: 5′-TGGATGGGCAAGGCAAGGTC-3′
	R: 5′-AAGTTGGGCACGGAAGCAGG-3′
lncARSR (Mmu)	F: 5′-TTTGAAATGCTCTTTGAGGGAT-3′
	F: 5′-TGCAGGTTGTCTGAAGTTGGA-3′
IRS2 (Humo)	F: 5′-CAAGAGCCCTGGCGAGTACA-3′
	R: 5′-CCGCGGATGCCAGTAGTG-3′
IRS2 (Mmu)	F: 5′-ATATTGCTGAAGAGCTTGGCG-3′
	R: 5′-TGTATGCGGTGCTCCGGGAAG-3′
GAPDH (Humo)	F: 5′-GGTCTCCTCTGACTTCAACA-3′
	R: 5′-GTGAGGGTCTCTCTCTTCCT-3′
GAPDH (Mmu)	F: 5′-GTTGTCTCCTGCGACTTCA-3′
	R: 5′-GCCCCTCCTGTTATTATGG-3′

*RT-qPCR* reverse transcription quantitative polymerase chain reaction, *lncARSR* (*Humo*), lncRNA regulator of AKT signaling associated with HCC and RCC (human); *lncARSR* (*Mmu*), lncRNA regulator of AKT signaling associated with HCC and RCC (house mouse), *IRS2* insulin receptor substrate 2, *GAPDH* glyceraldehyde-3-phosphate dehydrogenase

### Western blot analysis

Sufficiently ground liver tissues or HepG2 cells were lysed with radioimmunoprecipitation assay lysis buffer (C0481, Sigma-Aldrich), and total proteins were isolated. The proteins were then separated by 10% sodium dodecyl sulfate-polyacrylamide gel electrophoresis, and transferred onto a polyvinylidene fluoride membrane, which was blocked by 5% skimmed milk for 1 h. The membrane was incubated at 4 °C overnight with the diluted primary antibodies against YAP1 (1:1000, ab205270, rabbit), phosphorylated YAP1 (1:10,000, ab76252, rabbit), IRS2 (1:1000, ab134101, rabbit), AKT (1:10,000, ab179463, rabbit), phosphorylated AKT (1:5000, ab81283, rabbit), fatty acid synthase (Fasn, ab99359, 1:2000, rabbit), stearoyl-coenzyme A desaturase 1 (Scd1, ab19862, 1:1000, mouse), glycerol-3-phosphate acyltransferase (GPAT, ab69990, 1:5000, rabbit) and GAPDH (1:1000, ab8245, rabbit). All the above antibodies were purchased from Abcam Inc. (Cambridge, UK). Next, the membrane was incubated with horseradish peroxidase conjugated goat anti-rabbit immunoglobulin G (TransGen Biotech, Beijing, China), and developed with enhanced chemiluminescence solution (BaomanBio, Shanghai, China). The relative expression was described as the ratio of gray value of the target band to that of GAPDH band, which was analyzed by the image analysis software Image J.

### Nile red staining

The lipid accumulation of HepG2 cells was examined by staining with the lipophilic dye Nile Red (Sigma-Aldrich). In brief, cells were fixed with 4% paraformaldehyde for 10 min and incubated with Nile Red solution at a final concentration of 1 mg/L in phosphate buffer saline (PBS) for 20 min at 37 °C. Then the cells were mounted with Prolong^®^ Gold antifade reagent containing 4′-6-diamidino-2-phenylindole (DAPI; Invitrogen, Carlsbad, CA, USA) and examined by a fluorescent microscope.

### Histological analysis

Sections of liver were embedded in Tissue-Tek OCT Compound and frozen with carbon dioxide ice. Then, sections were stained with Oil-red-O/60% isopropyl alcohol solution (Thermo Fisher Scientific). After being rinsed with 60% isopropyl alcohol and distilled water, the sections were counterstained by hematoxylin for 4 min, and then observed under a Zeiss Axioplan 2 upright microscope (Carl Zeiss, Jena, Germany).

### RNA immunoprecipitation (RIP) and RNA pull-down

RIP and RNA pull-down were conducted following the methods as previously described [12]. EZ-Magna RIP RNA-Binding Protein Immunoprecipitation Kit (EMD Millipore, Billerica, MA, USA) was used for RIP, and Rneasy Mini Kit (Qiagen, Hamburg, Germany) was utilized to purify RNA in RNA pull-down.

### RNA-fluorescence in situ hybridization (FISH)

The expression and distribution of lncARSR and YAP1 in HCC cells were determined by FISH. Cells were cultured in a 24-well plate with 5 × 10^3^ cells/well, and penetrated with PBS containing 0.5% Triton X-100. The cells were then blocked with pre-hybridization solution at 37 °C and hybridized with lncARSR probe at 37 °C overnight in dark. Next, the cells were washed with FISH solution at 42 °C and stained with DAPI. The FISH signals were detected using the tyramide signal amplification system (PerkinElmer Corporation, Norwalk, CT, USA) and analyzed with a fluorescence microscope (IX70, Olympus Medical Systems Co., Tokyo, Japan).

### 3-(4, 5-dimethylthiazol-2-yl)-2, 5-diphenyltetrazolium bromide (MTT) assay

Cell proliferation was examined by a MTT cell proliferation kit (Cell Biolabs Inc, San Diego, CA, USA) following the manufacturer’s instruction.

### Soft-agar colony formation

HepG2 cells (0.5 × 10^6^) were suspended in 8 mL of 0.4% top agar (Sigma-Aldrich), cultured in a 6-cm petri dish in 2 × DMEM supplemented with 20% FBS and wrapped with 3.5 mL of 0.7% bottom agar. After 14 days, the number of cell colonies was counted in three randomly selected regions from each plate.

### Transwell invasion assay

The in vitro transwell invasion assay was conducted in 24-well plates using transwell chambers (with 8-μM diameter; Corning Incorporated, Corning, NY, USA). Transwell chambers pre-coated with Matrigel were pre-supplemented with 600 μL DMEM containing 20% FBS at 37 °C for 1 h. The transwell basolateral chamber was also supplemented with DMEM containing 20% FBS. After 48 h of transfection, HepG2 cells were resuspended with DMEM containing 10% FBS. Cells were cultured in the apical chamber at 37 °C with 5% CO_2_ for 24 h, and then the cells in intimal microporous film were scrubbed off using a cotton swab. Cells were fixed with 4% paraformaldehyde and stained with 0.1% crystal violet. The stained cells were observed under an inverted microscope and quantified.

### Flow cytometry

HepG2 cells 24 h before transfection were seeded into a 6-well plate. After 48 h transfection, cells were fixed with 70% ethanol. Then, cells were resuspended in PBS and incubated with RNase (100 μg/mL) and propidium iodide (60 μg/mL; Sigma-Aldrich). Cells were subsequently sorted by the FACSCalibur System (BD Biosciences, San Jose, CA, USA) and the cell cycles were analyzed by CellQuest software. Proliferation index (PI) was calculated as PI = (S + G2/M)/G1, in which S, G2/M and G1 referred to the percentage of cells in S, G2/M and G1 phase, respectively.

### Xenograft tumor assay

HepG2 cells were placed into a 6-well plate and transfected with shRNA against NC or shRNA targeting lncARSR. After 24 h, 5 × 10^5^ cells resuspended in 0.1 × PBS were hypodermically injected into the right side of the back of athymic nude mice (n = 15). Tumors were observed and measured every 3 days, and the tumor volume (cm^3^) was calculated as d^2^ × D/2, in which d was the shortest diameter and D was the longest diameter. When the diameter of tumors reached 1.5 cm, the mice were euthanized, and tumors were removed and weighed.

### Statistical analysis

The data were processed using SPSS 21.0 statistical software (IBM Corp., Armonk, New York, USA). Then data distribution was tested for normality and homogeneity of variance. Data were expressed as mean ± standard deviation. If departure from normality and variance was not observed, unpaired *t*-test was used for analysis between 2 experimental groups, while one-way analysis of variance (ANOVA) was employed for comparison among multiple groups. Repeated measures ANOVA were utilized to compare data among multiple groups at different time points. Pairwise comparison within group was examined by post hoc test. If departures from normality or variance were found, rank-sum test was conducted. *p* < 0.05 was considered statistically significant.

## Results

### LncARSR expression is increased in both high-fat-fed mice and HepG2 cells treated with high fatty acid

**Fig. 1:**
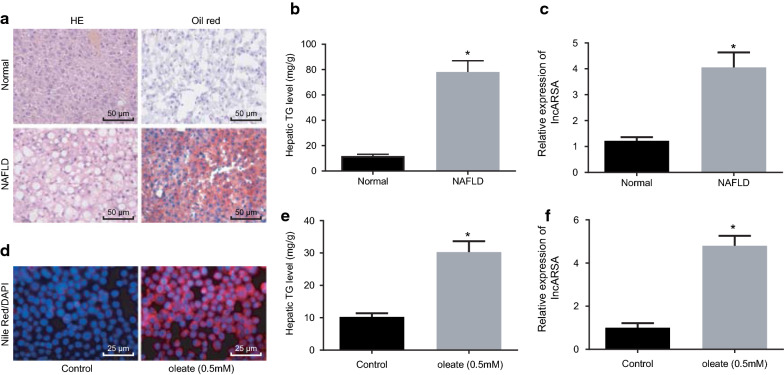
Highly-expressed lncARSR is found in NAFLD mice and HepG2 cells with oleate. **a** Fat accumulation in liver from the HFD-fed mice detected by H&E staining and Oil Red-O staining (×200) (n = 15). **b** TG content in liver from HFD-fed mice (n = 15). **c** LncARSR level in liver (n = 15) examined by RT-qPCR. **d** Fat content in HepG2 cells treated with 0.5 nM oleate monitored by Nile Red staining. **e** TG content in oleate-treated HepG2 cells (×400); **f** lncARSR expression in HepG2 cells treated with oleate determined by RT-qPCR. All the data were measurement data and described as mean ± standard deviation. Differences between two groups were analyzed by unpaired *t*-test. The experiment was conducted three times. *p < 0.05 against normal diet-fed mice or control HepG2 cells

It was reported that over-expressing lncARSR accelerated the accumulation of liver fat in vivo and in vitro, while silencing lncARSR led to reduction of liver fat, which suggested that lncARSR may participate in regulation of liver fat in NAFLD [7]. To further explore the role of lncARSR in NAFLD, we generated NAFLD mouse models by feeding C57Bl/6 mice with HFD. First, accumulation of lipid in liver of NAFLD mice was detected, which displayed that fat accumulation in NAFLD mice was significantly higher than that in normal mice (Fig. 1a). Meanwhile, the content of TG in liver from NAFLD mice was also obviously enhanced (Fig. 1b), suggesting the successful establishment of the NAFLD model. LncARSR expression was determined in liver, which exhibited that lncARSR expression was upregulated in liver from NAFLD mice (Fig. 1c).

Subsequently, HepG2 cells were treated with 0.5 mM oleate. It was observed that oleate treatment increased the lipid content (Fig. 1d) and TG content (Fig. 1e) in HepG2 cells. Next, lncARSR expression was examined, which showed that lncARSR expression was elevated in oleate-treated cells (Fig. 1f). Thus, lncARSR was expressed highly in NAFLD mice and oleate-treated HepG2 cells.

### LncARSR specifically binds to YAP1 and blocks YAP1 phosphorylation as well as promotes import of YAP1 into nucleus

**Fig. 2:**
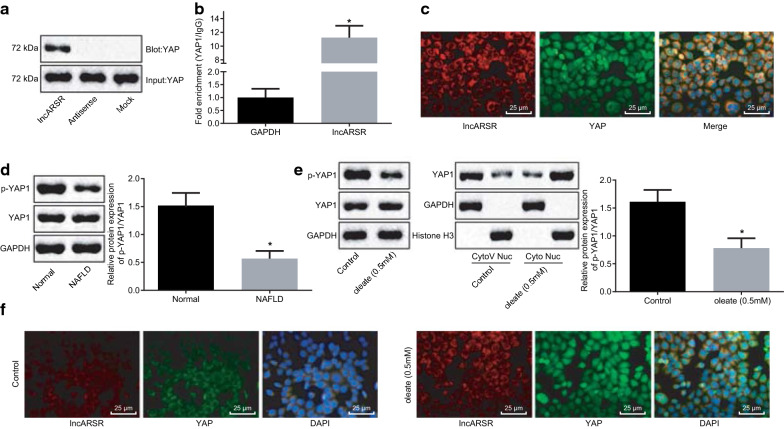
Specific binding between lncARSR and YAP1 inhibits YAP1 phosphorylation and accelerates YAP1 nuclear translocation. **a** Binding relationship between lncARSR and YAP1 detected by RNA pull down. **b** Interaction between lncARSR and YAP1 further verified by RIP assay. **c** Co-localization of lncARSR and YAP1 examined by RNA FISH (×400). **d** Levels of YAP1 and phosphorylated YAP1 in liver of NAFLD mice and normal mice determined by western blot analysis (n = 15). **e** Levels of YAP1 and phosphorylated YAP1 in nucleus and cytoplasm of HepG2 cells treated with oleate monitored by western blot analysis. **f** Co-localization between lncARSR and YAP1 in cells treated with oleate examined by RNA FISH (×400). All the measurement data were expressed as mean ± standard deviation. Comparisons among multiple groups were analyzed with one-way ANOVA, and the unpaired *t*-test was made for analysis between two groups. The experiment was repeated three times. *p < 0.05 against normal diet-fed mice or control HepG2 cells

To explore the binding of lncARSR and YAP1, we conducted RNA pull down assay, which revealed that the full-length lncARSR probe pulled down YAP1 (Fig. 2a). In order to further confirm the binding relationship, the RIP assay was carried out, which displayed the binding between lncARSR and YAP1 (Fig. 2b). Then, the subcellular localization of lncARSR and YAP1 was examined by RNA FISH, disclosing that lncARSR was co-localized with YAP1 in cytoplasm (Fig. 2c).

Next, the levels of YAP1 and phosphorylated YAP1 in NAFLD mice were determined. It was unraveled that phosphorylation level of YAP1 in liver from NAFLD mice was obviously lower than that from normal mice (Fig. 2d). Furthermore, the levels of YAP1 and phosphorylated YAP1 in nucleus and cytoplasm of HepG2 cells treated with oleate were examined, which revealed that phosphorylation of YAP1 was overtly decreased in cytoplasm of oleate-treated HepG2 cells, while the nuclear translocation of YAP1 was enhanced in oleate-treated HepG2 cells (Fig. 2e, f). Thus, lncARSR specifically interacted with YAP1 and promoted YAP1 nuclear translocation.

### LncARSR activates IRS2/AKT pathway by inhibiting YAP1 phosphorylation

**Fig. 3:**
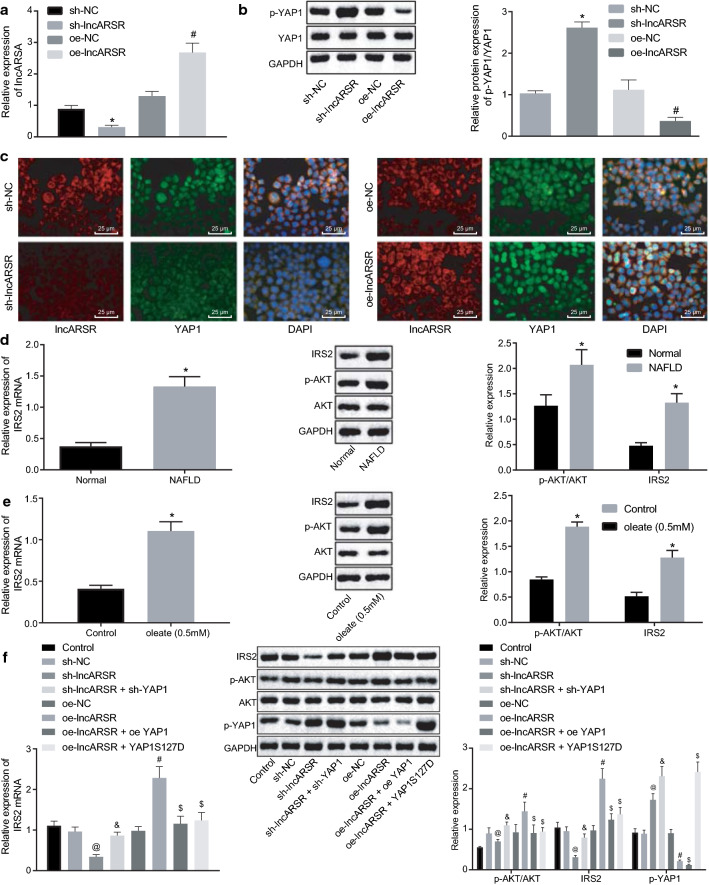
lncARSR participates in activation of the IRS2/AKT pathway by blocking YAP1 phosphorylation. **a** LncARSR expression after alteration of lncARSR measured using RT-qPCR. **b** YAP1 expression and phosphorylation of YAP1 after alteration of lncARSR assessed by western blot analysis. **c** Co-localization of lncARSR and YAP1 after alteration of lncARSR detected by RNA FISH (×400). **d** Expression of IRS2, AKT and phosphorylation of AKT in NAFLD mice determined by western blot analysis. **e** Levels of IRS2, AKT and phosphorylation of AKT in oleate-treated HepG2 cells examined by western blot analysis. **f** IRS2 expression and phosphorylation of AKT after alteration of lncARSR and YAP1 detected by western blot analysis. *p < 0.05 against normal or control treatments; ^@^*p* < 0.05 against sh-NC treatment; ^#^*p* < 0.05 against oe-NC treatment ^&^*p* < 0.05 against sh-lncARSR treatment; ^$^*p* < 0.05 against oe-lncARSR treatment. The measurement data were descried as mean ± standard deviation. Unpaired *t*-test was used for comparison between two groups; one-way ANOVA for analyzing multiple groups. Experiments were performed three times

Aiming to further investigate the effect of lncARSR on phosphorylation and nuclear translocation of YAP1, we over-expressed lncARSR in HepG2 cells. RT-qPCR revealed that oe-lncARSR treatment obviously increased lncARSR expression, but sh-lncARSR potently reduced lncARSR expression (Fig. 3a). Western blot analysis displayed that phosphorylated YAP1 expression was reduced by over-expressing lncARSR but was enhanced by silencing lncARSR (Fig. 3b).

Next, based on RNA-FISH, over-expressing lncARSR resulted in decreased distribution of YAP1 in cytoplasm but increased distribution of YAP1 in the nucleus (Fig. 3c), which was opposite after silencing lncARSR, indicating that lncARSR regulates YAP1 nuclear translocation.

YAP1, as the hub component of Hippo pathway, regulates the progression of liver cancer [13]. On the other hand, activation of the Hippo pathway prevented fatty liver and liver cancer by inhibiting IRS2/AKT pathway [11]. To further explore the involvement of IRS2/AKT in NAFLD, we measured IRS2 and AKT level in NAFLD mice and HepG2 cells treated with oleate using RT-qPCR and western blot analysis. In comparisons with normal mice, IRS2 expression was elevated both at mRNA and protein levels and the phosphorylation level of AKT was also upregulated in NAFLD mice (Fig. 3d). Consistently, IRS2 expression and phosphorylation level of AKT were also increased in oleate-treated HepG2 cells (Fig. 3e).

Furthermore, HepG2 cells were infected with oe-NC, sh-NC, oe-lncARSR, oe-lncARSR + sh-YAP1, sh-lncARSR, sh-lncARSR + oe-YAP1 or sh-lncARSR + YAP1S127D. Then the expression of IRS2 and AKT and the phosphorylation of AKT and YAP1 were determined. It was illustrated that IRS expression and phosphorylation level of AKT were elevated but phosphorylation level of YAP1 was diminished in HepG2 cells over-expressing lncARSR, while infection with oe-lncARSR + sh-YAP1 decreased IRS expression and phosphorylation level of AKT and YAP1. In addition, silencing lncARSR reduced IRS expression and phosphorylation level of AKT but increased phosphorylation level of YAP1 in HepG2 cells, which was further promoted by additional infection with YAP1S127D. However, IRS expression and phosphorylation level of AKT and YAP1 were enhanced in HepG2 cells infected with sh-lncARSR + oe-YAP1 (Fig. 3f). Hence, lncARSR promoted nuclear translocation of YAP1 to activate IRS2/AKT pathway.

### LncARSR increases lipid accumulation, cell proliferation and invasion of oleate-treated HepG2 cells

**Fig. 4:**
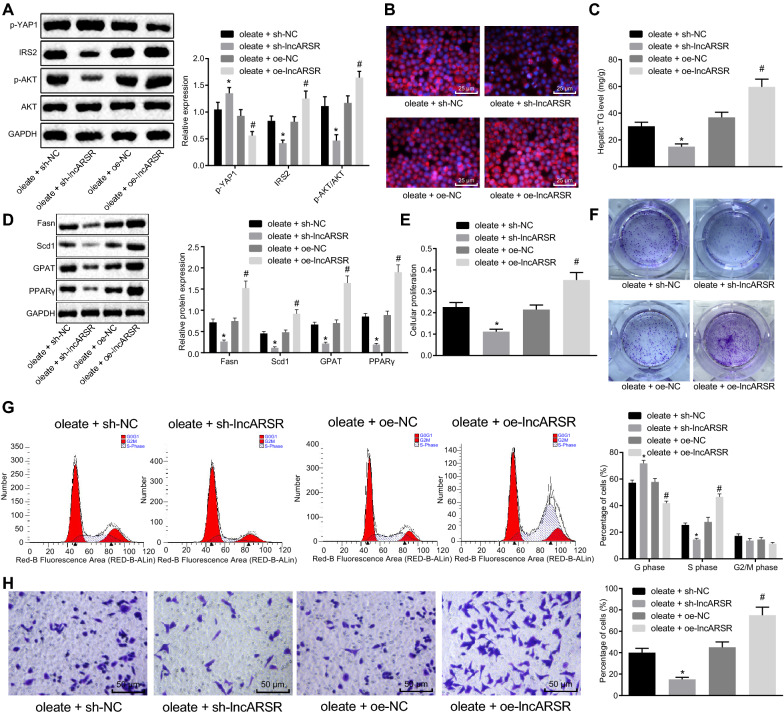
LncARSR increases cell proliferation, invasion, and cycle as well as lipid accumulation in oleate-treated HepG2 cells. Oleate-treated HepG2 cells were infected with lentivirus of sh-lncARSR, sh-NC, oe-lncARSR or oe-NC. **a** Expression of YAP1, IRS2, AKT and phosphorylation of AKT proteins in HepG2 cells. **b** Lipid accumulation in HepG2 cells (×400). **c** TG content in HepG2 cells. **d** Expression of adipogenesis related proteins (Fasn, Scd1 and GPA) and PPARγ in HepG2 cells. **e** Cell proliferation determined by MTT assay. **f** Cell proliferation monitored by soft-agar colony formation. **g** Cell cycle examined by flow cytometry. **h** Cell invasion inspected by transwell assay (× 200). *p < 0.05 against oleate-treated HepG2 cells treated with sh-NC; ^#^*p* < 0.05 against oleate-treated HepG2 cells treated with oe-NC. Measurement data were expressed as mean ± standard deviation from at least three independent repeated experiments. Unpaired *t*-test and one-way ANOVA were used for comparisons between or among two groups or multiple groups

To further explore the function of lncARSR in HepG2 cells, lncARSR was over-expressed or silenced in oleate-treated HepG2 cells. Western blot analysis showed that infection with oe-lncARSR decreased YAP1, increased IRS2 expression and phosphorylation level of AKT, and did not affected AKT expression in oleate-treated cells, which was opposite in oleate-treated cells infected with sh-lncARSR (Fig. 4a).

Lipid accumulation under different conditions was determined by Nile Red staining. Over-expressing lncARSR overtly increased while silencing lncARSR significantly reduced lipid accumulation and TG contents in oleate-treated cells (Fig. 4b).

Western blot analysis results (Fig. 4d) showed that expression of adipogenesis related proteins (Fasn, Scd1 and GPA) and peroxysome proliferator activated receptor (PPARγ) was in line with TG expression in the cells (Fig. 4c) examined by Nile Red staining.

Results of the MTT assay and soft-agar colony formation assay suggested that infection with oe-lncARSR distinctly increased while infection with sh-lncARSR visibly decreased proliferation of oleate-treated HepG2 cells (Fig. 4e, f). Flow cytometry documented that over-expressing lncARSR enhanced, while silencing lncARSR reduced, the number of oleate-treated HepG2 cells arrested in S phase (Fig. 4g).

Transwell assay revealed that there were more invasive cells after over-expressing lncARSR, but fewer invasive cells after silencing lncARSR in oleate-treated HepG2 cells (Fig. 4h). Therefore, lncARSR promoted lipid accumulation, cell proliferation, invasion and cycle in oleate-treated HepG2 cells.

### LncARSR silencing can inhibit lipid accumulation in HFD-fed mice

**Fig. 5:**
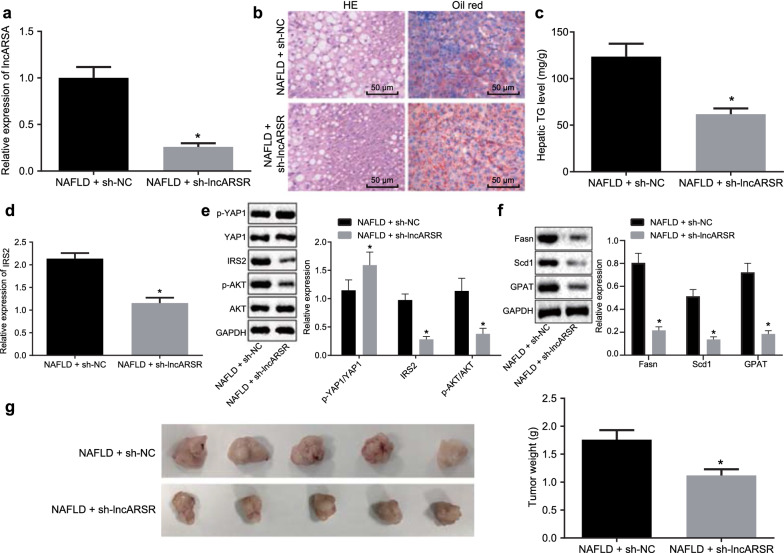
Silencing lncARSR alleviates lipid accumulation in HFD-fed mice and inhibits tumor formation in nude mice. HFD-fed mice were injected with lentivirus of sh-lncARSR or sh-NC. **a** LncARSR expression in mice detected by RT-qPCR. **b** Lipid accumulation in mice determined by H&E staining and Oil Red-O staining (×200). **c** TG content in mice. **d** IRS2 expression in mice detected by RT-qPCR. **e** Expression YAP1, IRS2 and AKT and phosphorylation of YAP1 and AKT levels detected by western blot analysis. **f** Expression of adipogenesis related proteins (Fasn, Scd1 and GPA) in mice detected by western blot analysis. HFD-fed mice were injected with HepG2 cells stably transfected with sh-lncARSR or sh-NC. **g** Tumor size and volume examined in xenograft tumor model. n = 15 *p < 0.05 against NAFLD mice injected with sh-NC lentivirus or sh-NC-transfected HepG2 cells. Differences between two groups were analyzed by unpaired *t*-test. Results were expressed as mean ± standard deviation

Furthermore, mice were fed with HFD and injected with lentivirus of sh-lncARSR. RT-qPCR, demonstrated that sh-lncARSR lentivirus declined lncARSR expression in NAFLD mice (Fig. 5a). Meanwhile, H&E staining and Oil Red-O staining depicted that lipid accumulation in NAFLD mice with sh-lncARSR lentivirus was decreased (Fig. 5b). Moreover, silencing lncARSR drastically reduced the TG content in livers of NAFLD mice (Fig. 5c).

Consistently, we also found out that silencing lncARSR in NAFLD mice obviously reduced mRNA level of IRS2 (Fig. 5d) and overtly increased phosphorylation of YAP1 (Fig. 5e). Next, expression of adipogenesis related proteins (Fasn, Scd1 and GPA) was assessed, which documented that in liver of NAFLD mice, Fasn, Scd1 and GPA expression was reduced after silencing lncARSR (Fig. 5f). On the other hand, to observe lncARSR effect on tumor growth, HFD-fed nude mice were injected hypodermically with HepG2 cells transfected with sh-lncARSR. Transfection with sh-lncARSR markedly reduced the volume of tumors in mice (Fig. 5g). In summary, lncARSR silencing alleviated NAFLD in mice.

## Discussion

NAFLD, which is characterized by lipid accumulation of the liver, is prevalent in around 25% of adults, especially in diabetic patients [14]. Normally, NAFLD is frequently related to obesity and HFD, and originates from the abnormal accumulation of TG in livers, which may finally develop to liver cancer or cirrhosis [15]. However, the pathogenesis of NAFLD still needs to be further explored. In a previous study, over-expressing lncARSR was reported to accelerate the accumulation of liver fat in vivo and in vitro, which suggested that lncARSR may participate in NAFLD and may function as a novel therapeutic target for NAFLD [7]. Based on these facts, this study was designed to explore the effects of lncARSR on NAFLD. The collected evidences derived from this study demonstrated that lncARSR silencing could alleviate NAFLD by inactivating IRS2/AKT pathway via YAP1.

Initially, increased lncARSR expression was found in oleate-treated HepG2 cells and HFD-fed mice, suggesting that lncARSR may be associated with the progression of NAFLD. LncARSR, as one of the up-regulated lncRNAs in NAFLD, was proved to promote liver cancer stem cell expansion and HCC differentiation [16]. Similarly, lncARSR was upregulated in HCC cells and promoted doxorubicin resistance of HCC cells by activation of the PI3K/AKT pathway [17]. The present study also found that lncARSR could specifically bind to YAP1 and promote YAP1 nuclear translocation through inhibiting its phosphorylation. The transcriptional activator YAP acts as a key regulator in the Hippo pathway [18]. In liver disease, YAP has also been reported to be increased with the increased degree of liver impairment [19]. Moreover, another study has reported that LATS2 modulated phosphorylation of YAP1 and regulated YAP1 in NAFLD [20]. Consistently, Qu et al. have also confirmed that lncARSR interacted with YAP and promoted YAP import into nucleus [12].

Furthermore, another important finding in this study was that lncARSR activated the IRS2/AKT pathway to elevate lipid accumulation in vivo and in vitro, accompanied by increased expression of Fasn, Scd1 and GPA, and accelerate NAFLD progression through inhibition of YAP1 phosphorylation. A previous study [21] revealed that the IRS2/PI3K/AKT pathway was activated in the liver of NAFLD models. LncARSR regulating AKT-related pathway was also exhibited in another study [17], in which lncARSR activates the PI3K/AKT pathway by promoting PTEN expression. Furthermore, Jeong et al. reported that Hippo-YAP/TAZ connecting with activation of IRS2/AKT plays a role in the development of NAFLD [22]. The previous study showed NAFLD was characterized by dysregulation of lipid metabolism in the liver, and less-differentiated HepG2 cells could suppress lipid accumulation [23]. Likewise, another study also explained that in the setting of NAFLD, lipid accumulation gives rise to liver damage and disease fibrosis [24]. Fasn and Scd1 play critical roles in hepatic fatty acid synthesis [25]. The treatment with GPA was identified to be associated with hepatic steatosis and lipid accumulation [26]. Reduction of Fasn and Scd1 expression was observed after alleviation of oleate-induced NAFLD [27]. Moreover, a study showed that depletion of lncARSR suppressed hepatic lipid accumulation in vivo and in vitro by decreasing Fasn and Scd1 expression via inactivation of the PI3K/AKT/mTOR pathway [7], which was in line with our results. Additionally, it is documented that the pathogenesis of NAFLD correlates to metabolic disorders such as lipid accumulation and insulin resistance [28]. Meanwhile, another study also showed promotion of insulin resistance and repression of hepatocellular glucose uptake in HepG2 cells treated with 0.5 mM oleate [29]. More importantly, the IRS2/AKT pathway was critical for liver insulin signaling to regulate insulin resistance in muscle and liver of diabetic rats [30]. However, although IRS2/AKT may also play a role in insulin resistance, it is not enough to resist the role of IRS2/AKT activation in promoting NAFLD and HCC. Therefore, the effect of lncARSR/YAP1/IRS2/AKT axis on NAFLD by regulating insulin resistance needs further elaboration.

## Conclusions

Overall, we conclude that lncARSR/YAP1 potentiates IRS2/AKT activity to promote NAFLD. Our results constitute significant new information to better understand the potential therapeutic effects of lncARSR via targeting YAP1 in NAFLD. The results also hold the great promise for illuminating the pathogenesis of NAFLD. However, further experiments in the future are needed by focusing the results to NAFLD or HCC alone, and expanding the results and discussion to elucidate the lncARSR function with more biological insights.

## Data Availability

The datasets used and/or analyzed during the current study are available from the corresponding author on reasonable request.

## Abbreviations

NAFLD: nonalcoholic fatty liver disease
HepG2: hepatocellular carcinoma cells
HFD: high fatty diet
RCC: renal cell cancer
YAP: yes-associated protein
GAPDH: glyceraldehyde-3-phosphate dehydrogenase
HCC: hepatocellular carcinoma

## References

[1] Wu H, Ng R, Chen X, Steer CJ, Song G (2016). MicroRNA-21 is a potential link between non-alcoholic fatty liver disease and hepatocellular carcinoma via modulation of the HBP1-p53-Srebp1c pathway. Gut.

[2] Calle EE, Kaaks R (2004). Overweight, obesity and cancer: epidemiological evidence and proposed mechanisms. Nat Rev Cancer.

[3] Chen X, Xu Y, Zhao D, Chen T, Gu C, Yu G (2018). LncRNA-AK012226 is involved in fat accumulation in db/db mice fatty liver and non-alcoholic fatty liver disease cell model. Front Pharmacol..

[4] Chen Y, Huang H, Xu C, Yu C, Li Y (2017). Long non-coding RNA profiling in a non-alcoholic fatty liver disease rodent model: new insight into pathogenesis. Int J Mol Sci.

[5] Takahashi K, Yan I, Haga H, Patel T (2014). Long noncoding RNA in liver diseases. Hepatology.

[6] Qu L, Ding J, Chen C, Wu ZJ, Liu B, Gao Y (2016). Exosome-transmitted lncARSR promotes sunitinib resistance in renal cancer by acting as a competing endogenous RNA. Cancer Cell.

[7] Zhang M, Chi X, Qu N, Wang C (2018). Long noncoding RNA lncARSR promotes hepatic lipogenesis via Akt/SREBP-1c pathway and contributes to the pathogenesis of nonalcoholic steatohepatitis. Biochem Biophys Res Commun.

[8] Lian I, Kim J, Okazawa H, Zhao J, Zhao B, Yu J (2010). The role of YAP transcription coactivator in regulating stem cell self-renewal and differentiation. Genes Dev.

[9] Jung KH, McCarthy RL, Zhou C, Uprety N, Barton MC, Beretta L (2016). MicroRNA regulates hepatocytic differentiation of progenitor cells by targeting YAP1. Stem Cells..

[10] Xin M, Kim Y, Sutherland LB, Qi X, McAnally J, Schwartz RJ (2011). Regulation of insulin-like growth factor signaling by Yap governs cardiomyocyte proliferation and embryonic heart size. Sci Signal..

[11] Jeong SH, Kim HB, Kim MC, Lee JM, Lee JH, Kim JH (2018). Hippo-mediated suppression of IRS2/AKT signaling prevents hepatic steatosis and liver cancer. J Clin Invest..

[12] Qu L, Wu Z, Li Y, Xu Z, Liu B, Liu F (2016). A feed-forward loop between lncARSR and YAP activity promotes expansion of renal tumour-initiating cells. Nat Commun..

[13] Tschuor C, Kachaylo E, Ungethum U, Song Z, Lehmann K, Sanchez-Velazquez P (2019). Yes-associated protein promotes early hepatocyte cell cycle progression in regenerating liver after tissue loss. FASEB Bioadv..

[14] Cicero A, Colletti A, Bellentani S (2018). Nutraceutical approach to non-alcoholic fatty liver disease (NAFLD): the available clinical evidence. Nutrients..

[15] Sun C, Liu X, Yi Z, Xiao X, Yang M, Hu G (2015). Genome-wide analysis of long noncoding RNA expression profiles in patients with non-alcoholic fatty liver disease. IUBMB Life.

[16] Yang C, Cai WC, Dong ZT, Guo JW, Zhao YJ, Sui CJ (2019). lncARSR promotes liver cancer stem cells expansion via STAT3 pathway. Gene.

[17] Li Y, Ye Y, Feng B, Qi Y (2017). Long Noncoding RNA lncARSR promotes doxorubicin resistance in hepatocellular carcinoma via modulating PTEN-PI3K/Akt Pathway. J Cell Biochem.

[18] Liu F, Lagares D, Choi KM, Stopfer L, Marinkovic A, Vrbanac V (2015). Mechanosignaling through YAP and TAZ drives fibroblast activation and fibrosis. Am J Physiol Lung Cell Mol Physiol.

[19] Chen P, Luo Q, Huang C, Gao Q, Li L, Chen J (2018). Pathogenesis of non-alcoholic fatty liver disease mediated by YAP. Hepatol Int..

[20] Guo C, Wang X, Liang L (2015). LATS2-mediated YAP1 phosphorylation is involved in HCC tumorigenesis. Int J Clin Exp Pathol..

[21] Xu H, Zhou Y, Liu Y, Ping J, Shou Q, Chen F (2016). Metformin improves hepatic IRS2/PI3K/Akt signaling in insulin-resistant rats of NASH and cirrhosis. J Endocrinol.

[22] Jeong SH, Lim DS (2018). Insulin receptor substrate 2: a bridge between Hippo and AKT pathways. BMB Rep..

[23] Ito S, Honda G, Fujino Y, Ogata S, Hirayama-Kurogi M, Ohtsuki S (2019). Knockdown of orphan transporter SLC22A18 impairs lipid metabolism and increases invasiveness of HepG2 Cells. Pharm Res.

[24] Ipsen DH, Lykkesfeldt J, Tveden-Nyborg P (2018). Molecular mechanisms of hepatic lipid accumulation in non-alcoholic fatty liver disease. Cell Mol Life Sci.

[25] Zhang J, Wang Y, Fu L, Feng YJ, Ji YL, Wang H (2018). Subchronic cadmium exposure upregulates the mRNA level of genes associated to hepatic lipid metabolism in adult female CD1 mice. J Appl Toxicol.

[26] Chaturvedi RK, Calingasan NY, Yang L, Hennessey T, Johri A, Beal MF (2010). Impairment of PGC-1alpha expression, neuropathology and hepatic steatosis in a transgenic mouse model of Huntington’s disease following chronic energy deprivation. Hum Mol Genet.

[27] Zhang J, Zhang SD, Wang P, Guo N, Wang W, Yao LP (2019). Pinolenic acid ameliorates oleic acid-induced lipogenesis and oxidative stress via AMPK/SIRT1 signaling pathway in HepG2 cells. Eur J Pharmacol.

[28] Kitade H, Chen G, Ni Y, Ota T (2017). Nonalcoholic fatty liver disease and insulin resistance: new insights and potential new treatments. Nutrients..

[29] Liu Y, Liao L, Chen Y, Han F (2019). Effects of daphnetin on lipid metabolism, insulin resistance and oxidative stress in OA treated HepG2 cells. Mol Med Rep..

[30] Okamoto MM, Anhe GF, Sabino-Silva R, Marques MF, Freitas HS, Mori RC (2011). Intensive insulin treatment induces insulin resistance in diabetic rats by impairing glucose metabolism-related mechanisms in muscle and liver. J Endocrinol.

