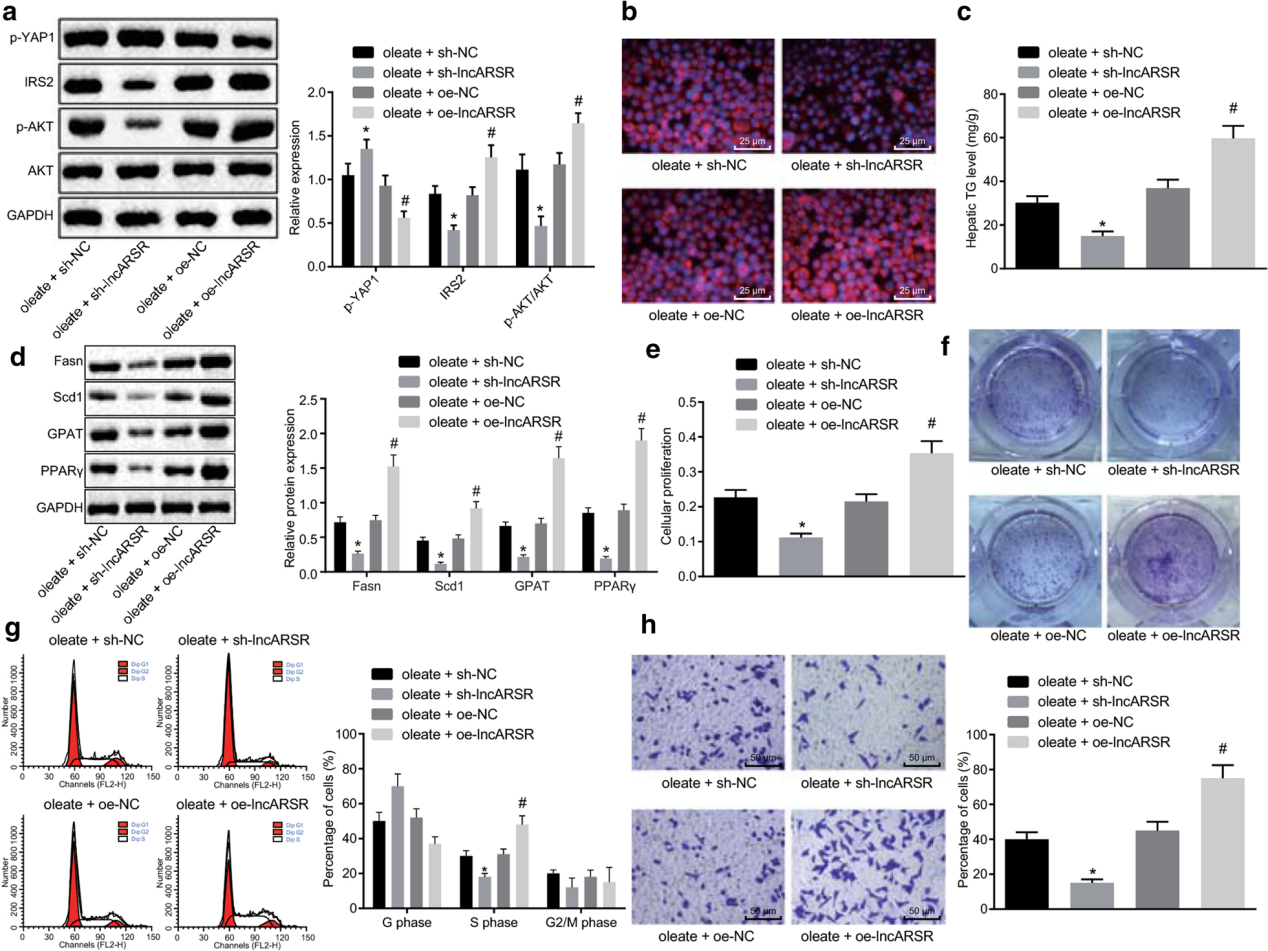# Correction to: Long noncoding RNA lncARSR promotes nonalcoholic fatty liver disease and hepatocellular carcinoma by promoting YAP1 and activating the IRS2/AKT pathway

**DOI:** 10.1186/s12967-021-02759-9

**Published:** 2021-10-19

**Authors:** Yuan Chi, Zheng Gong, He Xin, Ziwen Wang, Zhaoyu Liu

**Affiliations:** Department of Radiology, Shengjing Hospital of China Medical University, No. 36, Sanhao Street, Heping District, Shenyang, 110004 Liaoning People’s Republic of China

## Correction to: J Transl Med (2020) 18:126 10.1186/s12967-020-02225-y

Following publication of the original article [1], the authors identified an error in Fig. 4. The flow cytometry was used to detect the cycle changes of different groups of cells. The authors found that the results were biased due to the improper selection of parameters in flow cytometry. The flow cytometry results were carefully re-analyzed and corrected in Fig. 4g. The incorrect and correct figure are included in this Correction article. The original article has been updated.

**Correct Figure** **4:**

**Fig. 4:**
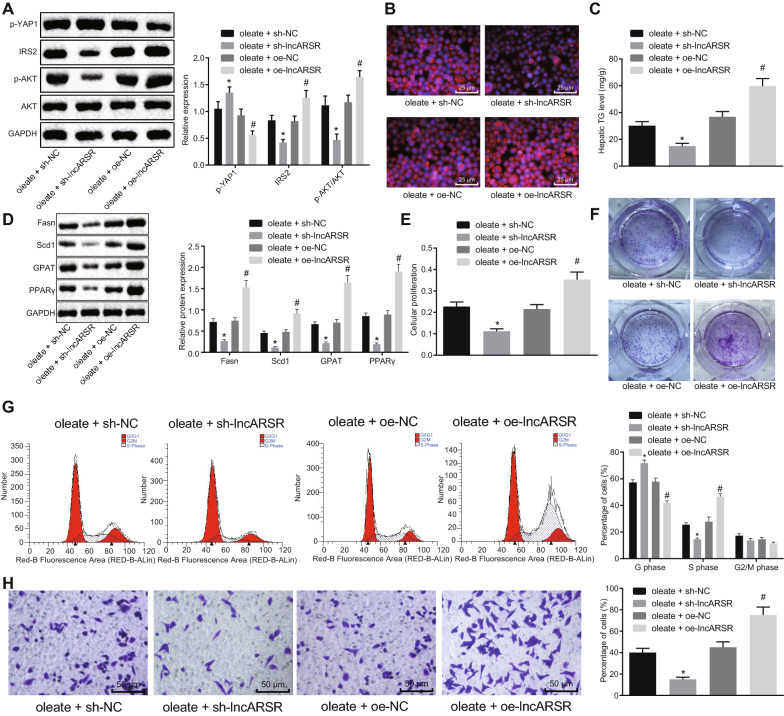
LncARSR increases cell proliferation, invasion, and cycle as well as lipid accumulation in oleate-treated HepG2 cells. Oleate-treated HepG2 cells were infected with lentivirus of sh-lncARSR, sh-NC, oe-lncARSR or oe-NC. **a** Expression of YAP1, IRS2, AKT and phosphorylation of AKT proteins in HepG2 cells. **b** Lipid accumulation in HepG2 cells (×400). **c** TG content in HepG2 cells. **d** Expression of adipogenesis related proteins (Fasn, Scd1 and GPA) and PPARγ in HepG2 cells. **e** Cell proliferation determined by MTT assay. **f** Cell proliferation monitored by soft-agar colony formation. **g** Cell cycle examined by flow cytometry. **h** Cell invasion inspected by transwell assay (× 200). ^*^p < 0.05 against oleate-treated HepG2 cells treated with sh-NC; ^#^*p* < 0.05 against oleate-treated HepG2 cells treated with oe-NC. Measurement data were expressed as mean ± standard deviation from at least three independent repeated experiments. Unpaired *t*-test and one-way ANOVA were used for comparisons between or among two groups or multiple groups

**Incorrect Figure** **4:**